# Gene biomarker prediction in glioma by integrating scRNA-seq data and gene regulatory network

**DOI:** 10.1186/s12920-021-01115-6

**Published:** 2021-12-04

**Authors:** Guimin Qin, Longting Du, Yuying Ma, Yu Yin, Liming Wang

**Affiliations:** School of Computer Science and Technology, Xidian University, Xi’an, 710071 China

**Keywords:** Glioma, Single-cell gene expression profile, Cell type, Tumor gene biomarkers

## Abstract

**Background:**

Although great efforts have been made to study the occurrence and development of glioma, the molecular mechanisms of glioma are still unclear. Single-cell sequencing technology provides a new perspective for researchers to explore the pathogens of tumors to further help make treatment and prognosis decisions for patients with tumors.

**Methods:**

In this study, we proposed an algorithm framework to explore the molecular mechanisms of glioma by integrating single-cell gene expression profiles and gene regulatory relations. First, since there were great differences among malignant cells from different glioma samples, we analyzed the expression status of malignant cells for each sample, and then tumor consensus genes were identified by constructing and analyzing cell-specific networks. Second, to comprehensively analyze the characteristics of glioma, we integrated transcriptional regulatory relationships and consensus genes to construct a tumor-specific regulatory network. Third, we performed a hybrid clustering analysis to identify glioma cell types. Finally, candidate tumor gene biomarkers were identified based on cell types and known glioma-related genes.

**Results:**

We got six identified cell types using the method we proposed and for these cell types, we performed functional and biological pathway enrichment analyses. The candidate tumor gene biomarkers were analyzed through survival analysis and verified using literature from PubMed.

**Conclusions:**

The results showed that these candidate tumor gene biomarkers were closely related to glioma and could provide clues for the diagnosis and prognosis of patients with glioma. In addition, we found that four of the candidate tumor gene biomarkers (*NDUFS5*, *NDUFA1*, *NDUFA13*, and *NDUFB8*) belong to the NADH ubiquinone oxidoreductase subunit gene family, so we inferred that this gene family may be strongly related to glioma.

## Background

Malignant tumors have a very large impact on human health due to their high mortality rate and high recurrence rate. There are many factors that affect tumorigenesis, including genetic variation, epigenetics, and external environmental influences. Glioma is the most common type of brain tissue tumor in complex diseases and accounts for approximately 40% of brain tissue tumors [1]. According to histological classification, glioma is an umbrella term used to describe the diffirent types of glial tumors: astrocytoma, oligodendroglioma, and glioblastoma and these three different tumors are derived from astrocytes, oligodendrocytes, and ependymal cells respectively. But now WHO classification is generally used. Glioma can be classified into 4 grades from WHO grade I to IV, with the higher WHO grade is, the more malignant the glioma is. The WHO grades of astrocytoma and oligodendroglioma are II or III. The WHO grade of glioblastoma is IV [2]. Therefore, It is of great importance to explore the molecular mechanisms of glioma, as these may help researchers develop glioma treatment strategies and drugs.

In recent years, many researchers have focused on the molecular mechanisms of glioma. Hu et al. constructed a coexpression network by calculating the differentially expressed genes (DEGs) between 971 glioma samples and 102 normal samples, and functional and pathway enrichment analyses indicated that the p53 signaling pathway and the pathway of neuroactive ligand-receptor interaction may play important roles in the progression of glioma, and three genes (*PUS7*, *EFR3B* and *NRCAM*) were potential biological agent landmarks [3]. Niu et al. used protein–protein interaction networks to screen key DEGs and then applied machine learning methods to reveal the molecular mechanisms of glioma [4]. Wang et al. integrated gene interaction information into a weighted random survival forest method to perform an accurate survival prediction and to discover a survival biomarker for glioma [5]. Zhou et al. identified the glioma-specific protein interaction network based on bulk RNA-seq data and performed enrichment analysis to verify disease-specific molecular complexes [6]. Due to the complexity of glioma, more genetic markers need to be discovered.

Recent advances in microfluidic technology have made it possible to isolate a large number of cells, and single-cell sequencing (scRNA-seq) data analysis has become one of the most noteworthy technical fields in bioinformatics [7–9]. The resolution of scRNA-seq technology is accurate to a single cell, can resolve more subtle differences among cells and is widely used in biology, including development [10, 11], infectious diseases [12, 13], immunity [14, 15], neurology [16] and oncology [17–21]. Cell type identification and/or rare cell type prediction based on scRNA-seq data can deepen the understanding of tumors and analyze the process of tumor occurrence [22]. At present, many methods have been proposed to identify cell types. For example, Kiselev et al. proposed a method for consistent clustering of single cells [23]. Wang et al. proposed Single-cell Interpretation via Multi-kernel LeaRning (SIMLR), which is based on single-cell data and multicore learning similarity measures. They used downscaling and clustering to analyze cell types [24]. Kim et al. implemented a semi-supervised learning classification tool, scReClassfy, to fine tune cell type annotations generated using any method in single-cell sequence datasets [25]. Lin et al. adopted an implicit missing value processing method to reduce the impact of dropout values in scRNA-seq data and achieved rapid and accurate cell type identification [26]. Grun et al. designed the RaceID method to identify rare cell types in complex single-cell populations through k-means and outlier detection methods [27]. Most of these methods directly identified cell types based on single-cell gene expression data without integrating multi-omics data.

In addition, gene expression levels are affected by a variety of regulatory factors, and it is also crucial for the treatment and prevention of complex diseases to understand the disturbance of transcriptional regulatory relationships. In terms of regulatory mechanisms, a transcription factor (TF) is a key gene regulatory factor that mainly activates or inhibits gene expression during the transcriptional stage. TFs participate in many important cellular processes, such as cell proliferation and cell differentiation. These cellular processes may affect the development of many complex diseases, including tumors [28]. For example, Zhang et al. reconstructed a multilayer signaling network that contains pathways from intercellular ligand-receptor interactions, intracellular TFs and their target genes. In this way, they discovered a new multilayer network biomarker (MNB) that was indicated to be valuable for the prognosis and prediction of glioma patients [29].

To further analyze the molecular mechanisms of glioma, in this study, we identified multiple cell types and candidate tumor gene biomarkers in glioma by integrating scRNA-seq data and transcriptional regulation pairs. Through gene enrichment analysis, survival analysis and PubMed analysis, our results showed that our method has an effective performance and provides clues for the diagnosis and prognosis of patients with glioma.

## Methods

### Materials

#### Single-cell gene expression data of glioma

To explore the molecular mechanisms of glioma, we downloaded the single-cell gene expression data with EXP0062 from the CancerSEA database [30]. The data contain a single-cell gene sequencing profile of 4043 tumor malignant cells, in which all malignant cells were derived from six glioma samples, and the tissue source of the samples was oligodendrocytes from cerebral cortex. More specific information about the dataset can be founded in [31]. The sample IDs are MGH36, MGH53, MGH54, MGH60, MGH93 and MGH97, respectively. The CancerSEA database uses methods such as copy number variation inference on the original single-cell data to ensure that all cells in the data set are tumor malignant cells.

#### Transcriptional regulation pairs

Gene transcriptional regulation pairs were collected from the HTRIdb [32] and TRRUST [33] databases. For HTRIdb, we collected 51,871 regulation pairs, and for TRRUST, we collected 8427 regulation pairs. The regulation pairs were the pairs between TFs and the regulatory targets (TARGETs). TARGETs contain target genes and target TFs. Therefore, we divided the regulation pairs into TF–TF pairs and TF-gene pairs, according to whether a TARGET is a TF or gene. Finally, we obtained 952 TFs, 17,600 target genes, 5694 TF-TF pairs and 53,408 TF-gene pairs.

#### Known glioma-related genes

We collected known cancer-related genes from the Online Mendelian Inheritance in Man (OMIM) [34] and the Catalogue Of Somatic Mutations In Cancer (COSMIC) [35] databases. OMIM is an authoritative database focusing on the relationship between disease phenotypes and genotypes and contains cancer-related genes with high confidence. COSMIC is a comprehensive somatic mutation database that contains thousands of somatic mutation information related to cancer development. In addition, we obtained known cancer-related genes from Bailey’s research results [36]. This research uses 26 different bioinformatics tools to analyze somatic mutations in a variety of cancers and provides services for cancer research. In total, we obtained 77 KGGs.

#### Bulk RNA-seq of gene expression data and clinical data from glioma

Bulk RNA-seq of gene expression data and clinical data from glioma were obtained from The Cancer Genome Atlas (TCGA) [37]. The clinical data contained overall survival (OS) data. To analyze the data more effectively, we retained samples that had a tissue type of oligodendrocytes only. In this way, the tissue type of the samples in bulk RNA-seq data was consistent with the tissue type of the samples in the single-cell gene expression data. In the end, we obtained 198 glioma samples. Then, to improve the data quality, we deleted genes whose expression values were less than 1 in more than half of the samples. Finally, to mitigate the influence of different samples on the expression level and avoid the influence of overcapturing features with extreme values and outliers, the z-score was used to normalize the gene expression values in the bulk RNA-seq expression data.

### Preprocessing of single-cell gene expression data

In the single-cell gene expression data, there are significant differences in tumor malignant cells among different patient samples. To comprehensively analyze tumor characteristics, we first explored the expression status of malignant cells in a single sample. Therefore, the original single-cell gene expression data were split according to the sample source to obtain multiple single-sample single-cell gene expression data.

Next, we cleaned the single-sample single-cell gene expression data from the perspective of cells and genes. First, the number of cells and genes were fitted to a normal distribution, and cells with significantly fewer expressed genes were deleted (FDR < 0.05). Then, the genes that had an expression value detected in at least 3 cells and had an average normalized expression value greater than 10^−5^ were retained. To effectively improve the signal-to-noise ratio, the genes affected by technical noise were ignored. We performed the M3Drop feature selection method [38] and obtained the feature genes of single-sample single-cell gene expression data with FDR < 0.01. Finally, we normalized each expression data with a logarithmic function of offset 1.

After preprocessing, we obtained a total of 6 single-sample single-cell gene expression data. In MGH36, there were 694 cells and 4608 feature genes. In MGH53, there were 726 cells and 4126 feature genes. In MGH54, there were 1174 cells and 4732 feature genes. In MGH60, there were 428 cells and 3609 feature genes. In MGH93, there were 440 cells and 3879 feature genes. In MGH97, there were 582 cells and 4113 feature genes.

### Identification of tumor consensus genes

Differences among samples of tumor malignant cells may affect the identification of genetic markers, so we first analyzed each single-sample single-cell gene expression data. First, we performed PCA on each single-sample single-cell gene expression data to determine the appropriate principal components. To prevent excessive capture of certain genes with large values, the z-score was used to normalize gene expression data. In addition, the criteria for determining the number of principal components were as follows: (1) the cumulative contribution rate was greater than 90%, and (2) the difference between two consecutive principal components was less than 0.1%. We used the minimal number in the numbers obtained from condition (1) and condition (2) as the final number of principal components.

Then, we adopted the idea of the k-nearest neighbors to construct a cell-specific network within a single sample. The Euclidean distance was used to calculate the distance between all cell pairs. The k-nearest neighbor relationships were determined for each cell, and the similarity between the two cells was calculated by Jaccard coefficients. Next, Louvain clustering [39] was used to achieve the initial division of cells in a single sample. The results of the initial division helped to analyze the expression status of malignant cells in a single sample. We constructed a cell-specific network with k as 20 and used the Seurat package [40] to complete the clustering process. According to each cell cluster in the initial division of each sample, the cells were divided into the cells belonging to the cell cluster and the remaining cells. We then performed the Limma package [41] to calculate the DEGs for each sample (lg2 | FC | > 1, *p* value < 0.05).

If a gene was a differential gene in multiple samples, the gene reflected the coexpression pattern among samples to some extent. We selected tumor consensus genes by screening the genes that were differentially expressed in at least 2 samples.

### Identification of tumor cell types

Each TF in the specific regulatory network was used to form its corresponding regulation meta module (RMM). Each RMM included all target genes and other TFs directly regulated by the core TF. Then, based on the entire single-cell gene expression data that contained all cells from different samples, the RMM was regarded as a new feature of malignant cells to construct a specific regulation expression matrix, in which the feature value was calculated by the Cell Score Method [42]. Then, hybrid clustering was used to identify the glioma cell types based on the matrix. The canopy clustering algorithm [43] was first performed on all malignant cells to provide the k value and initial clustering center for k-means clustering. Then, k-means clustering was used to identify cell types. The Calinski–Harabaz (CH) coefficient was used to tune the parameters of the hybrid clustering, and the larger the CH value was, the better the clustering results.

In the identified cell types, we combined the entire single-cell gene expression data after M3Drop feature selection and divided all cells into two groups for each cell type: the cells that belonged to the cell type and the remaining cells. The ROTS method [44] was performed to obtain the gene biomarkers for each cell type.

### Gene set enrichment analysis

To further analyze the functional characteristics of cell types, GO functional enrichment and pathway enrichment analyses were conducted for gene biomarkers from these cell types. We applied the Metascape tool [45] for enrichment analysis, which mainly provided five forms of gene annotations, including GO biological processes, Kyoto Encyclopedia of Genes and Genomes (KEGG) pathway, Reactome pathway database, Reactome canonical pathways and CORUM.

## Results

### Overview of the computational framework

**Fig. 1:**
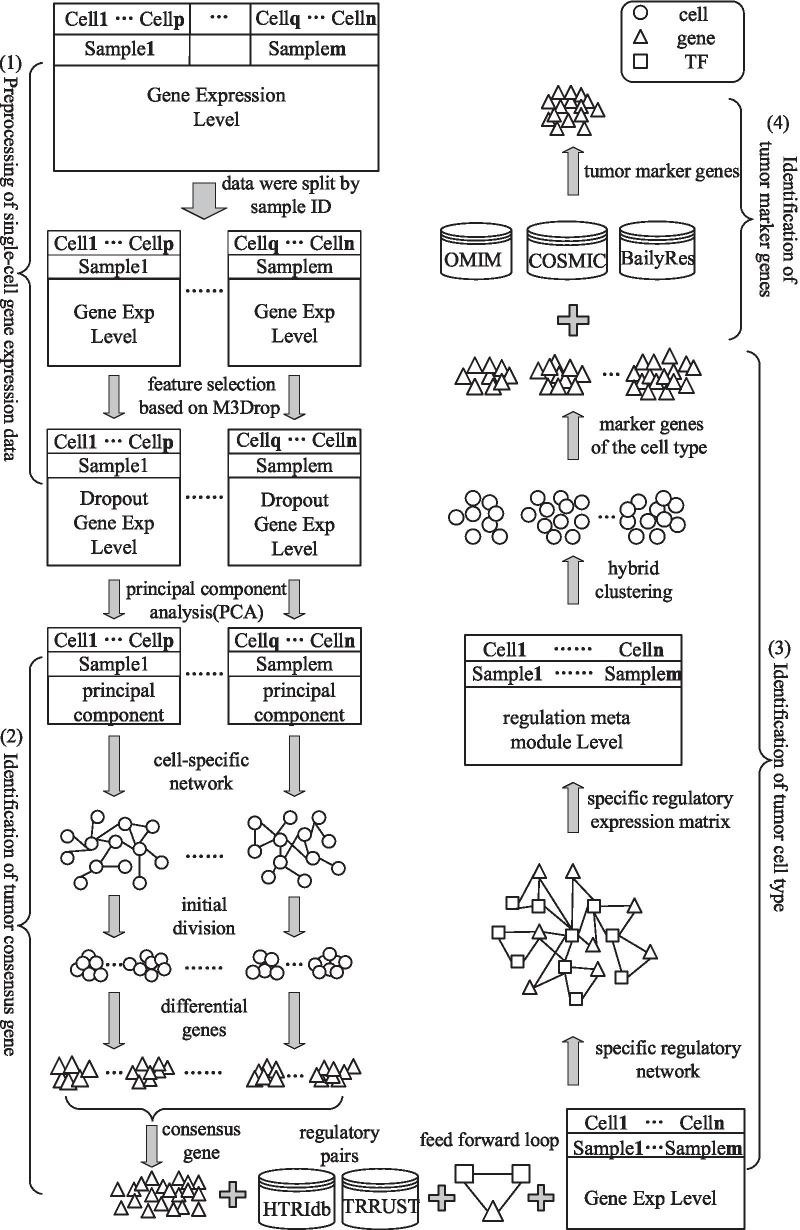
Overview of the computational framework. This framework consists of four steps: (1) Preprocessing of single-cell gene expression data. Split the original gene expression data according to the sample ID and then preprocess the data through data cleaning, feature selection and standardization. (2) Identification of tumor consensus genes. For each single-sample single-cell gene expression data, explore the gene expression patterns of all malignant cells. Then, tumor consensus genes were identified based on the overlapping degree of the differential genes among samples. (3) Identification of tumor cell types. Build a specific regulatory network based on tumor consensus genes and FFLs. Then, the single-cell specific regulatory expression matrix was constructed, and the cell types of glioma is obtained through a hybrid clustering method. (4) Identification of candidate tumor gene biomarkers. The tumor gene biomarkers were identified according to the degree of correlation between the candidate genes and the tumor eigenvector

We proposed a computational framework, which consisted of four steps (Fig. 1), to gain insight into the molecular mechanisms of glioma.

**Step 1** Preprocessing of single-cell gene expression data. The original single-cell gene expression data were split according to the sample ID. Then, we preprocessed the gene expression data through data cleaning, feature selection and standardization.

**Step 2** Identification of tumor consensus genes. For each single-sample single-cell gene expression data, we explored the gene expression patterns of all malignant cells through principal component analysis (PCA), cell-specific network construction, and differential gene identification. Then, based on the overlapping degree of the differential genes among samples, tumor consensus genes were identified.

**Step 3** Identification of tumor cell types. We combined the gene expression profiles of each sample and integrated transcriptional regulatory pairs. As a result, a specific regulatory network was built based on tumor consensus genes and feed forward loops (FFLs). Finally, the single-cell specific regulatory expression matrix was constructed, and a hybrid clustering method was used to obtain the cell types of glioma.

**Step 4** Identification of candidate tumor gene biomarkers. The gene biomarkers of the cell types were regarded as candidate genes, and then the tumor eigenvector was calculated by known glioma-related genes (KGGs). Finally, the tumor gene biomarkers were identified according to the degree of correlation between the candidate genes and the tumor eigenvector.

### Differential analysis of single-cell gene expression data among samples

We performed data cleaning and feature selection on the single-cell gene expression data, and then t-distributed stochastic neighbor embedding (TSNE) [46] was used to visualize the malignant cell clusters.

**Fig. 2:**
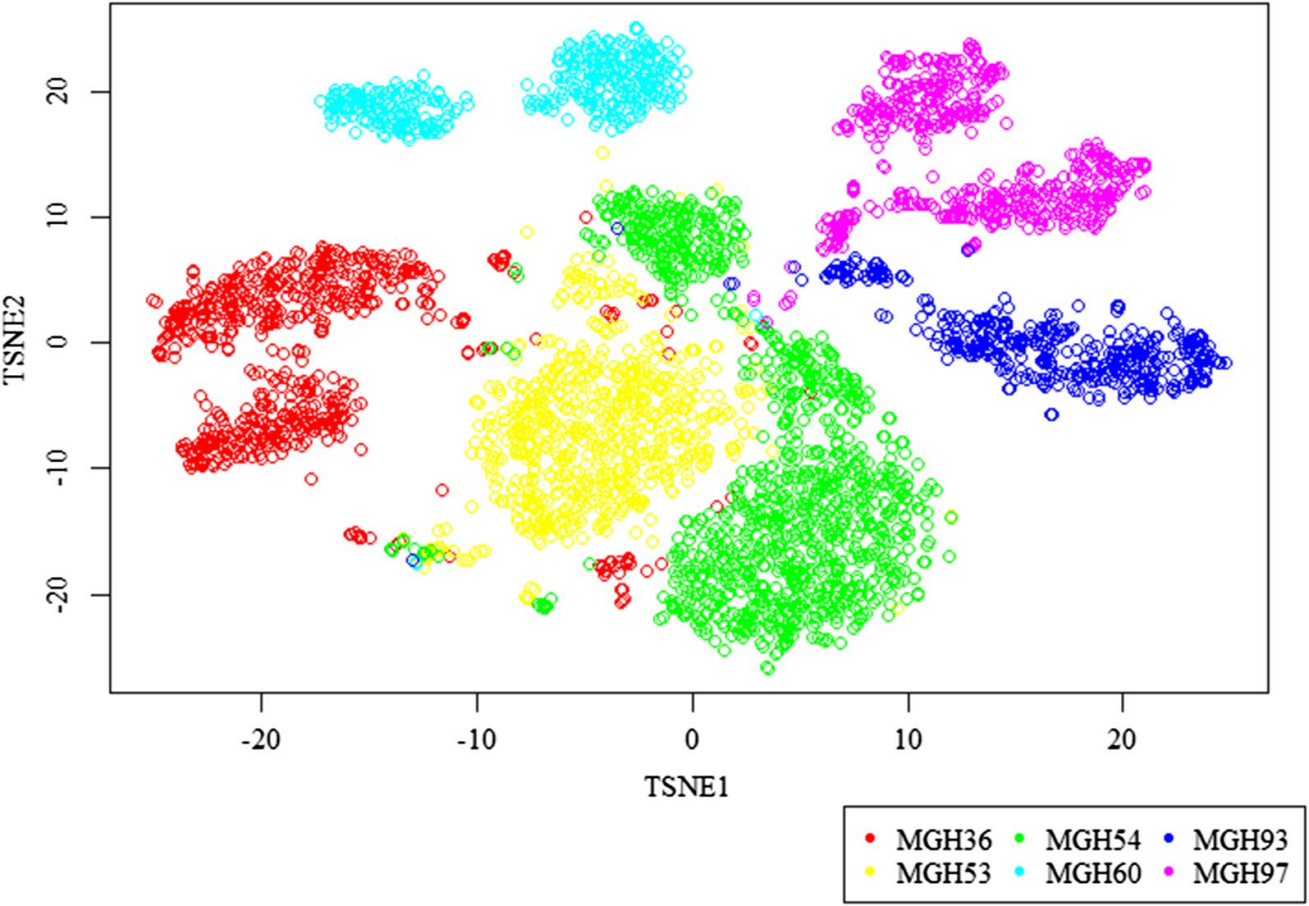
TSNE analysis of the entire single-cell gene expression data. Each point represents a cell and each color represents a tumor sample

Each point in Fig. 2 represents a cell, and each color represents a tumor sample. All of the tumor malignant cells were clustered according to their tumor sample source, and there were almost no mixing results of multiple tumor sample cells, which was consistent with previous studies [42, 47, 48]. Thus, there were considerably significant differences in malignant cells among samples, which inspired us to conduct our analysis at the sample level.

### Analysis of consensus genes

For each sample, the k-nearest neighbors and Jaccard coefficient were used to construct a cell-specific network, then Louvain clustering [39] was used to obtain the initial division of all cells, and the differentially expressed genes (DEGs) were calculated (Table 1). Finally, consensus genes were identified based on the overlapping degree of differential genes among different samples. In this paper, a total of 1123 tumor consensus genes were conservatively conserved by screening differential genes that were present in at least two samples.

**Table 1:** Cell-specific network of single-sample

Sample ID	Cell-specific network		Initial division*	Num. of DEGs
	Num. of nodes	Num. of edges		
MGH36	694	20,452	6	1206
MGH53	726	21,324	8	661
MGH54	1174	35,915	8	924
MGH60	428	15,271	5	545
MGH93	440	12,695	5	443
MGH97	582	19,057	6	607

*The number of clusters through Louvain clustering algorithm

**Fig. 3:**
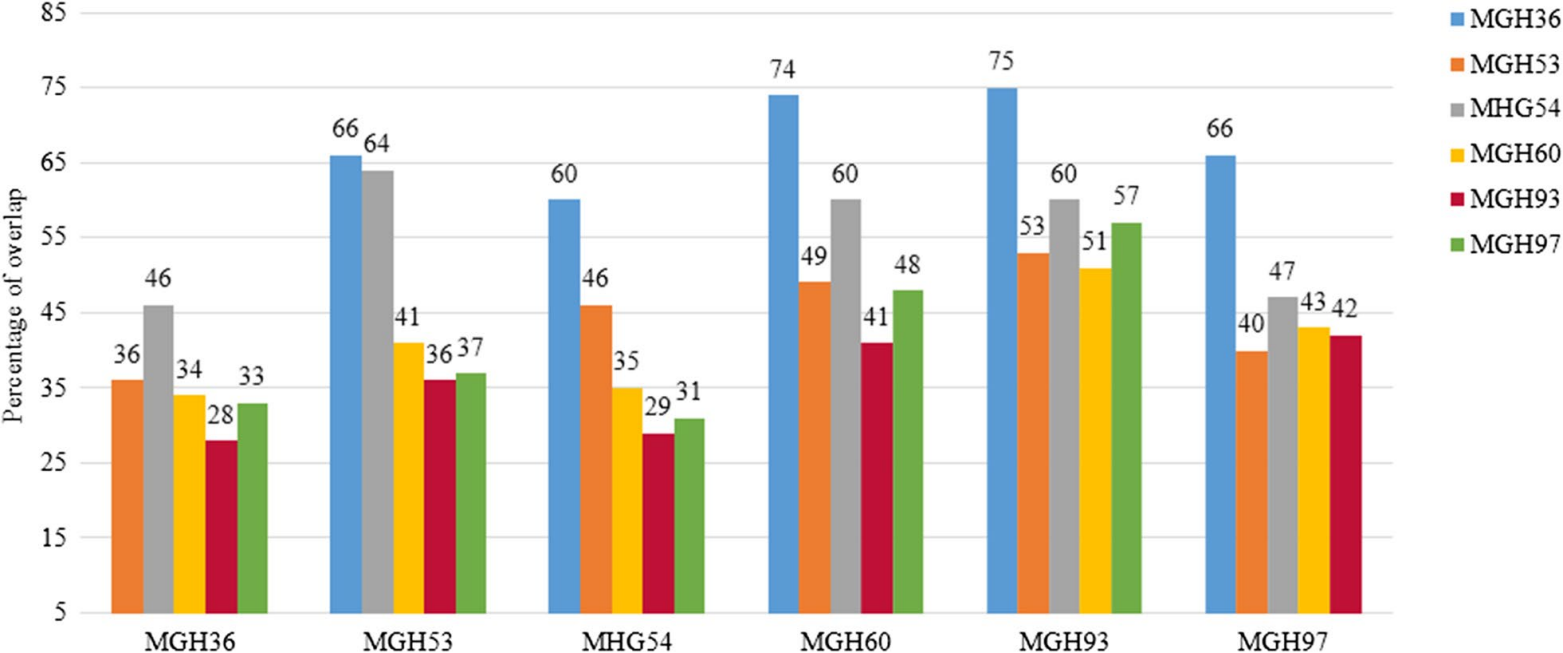
Overlap of differentially expressed genes between samples. The horizontal axis represents the sample source of all malignant cells in the glioma single-cell gene expression data, a total of 6 tumor samples. For each sample, tumor consensus genes, the differentially expressed genes that overlap with the other 5 samples, was founded. The vertical axis represents the proportion of tumor consensus genes in all the differentially expressed genes of the sample

To show that the overlapping degree of DEGs could describe the co-expression patterns of genes among different samples, we counted the overlapping degree of the DEGs in a certain sample and the other samples (Fig. 3). The x-axis represents the sample source of all malignant cells. For each sample, the DEGs that overlapped with the other 5 samples were calculated, respectively, and the y-axis represents the proportion of the overlapping DEGs in all the DEGs of the sample. Figure 3 shows that the highest percentage of overlap was 75%, the lowest percentage of overlap was 28%, and more than half of the overlap percentages were from 30 to 50%. The analysis results showed that the DEGs in different samples had a high degree of consistency, which further showed that the consensus genes reflected the common gene expression patterns in different samples.

### Specific regulatory network analysis

TF-TF pairs and TF-gene pairs were obtained from the Human Transcriptional Regulation Interactions database (HTRIdb) [32] and Transcriptional Regulatory Relationships Unraveled by Sentence-based Text mining (TRRUST) [33], and we constricted the target genes as tumor consensus genes. To improve the specificity of the regulatory network, the entire single-cell gene expression data containing all cells from all samples were used to adjust the network links. We first reassigned the missing values in the entire single-cell gene expression data using the method proposed by Venteicher et al. [49]. The new value of *E*_*i,j*_ was proportional to the expected expression of gene *i* in cell *j*, which was calculated by the average expression of gene *i* and the complexity of cell *j* (the number of detected genes). Then, we calculated the Euclidean distance for each regulation pair based on the entire expression data, and the maximum and minimum normalization was used to shrink the range of distance. We then calculated the similarity of the regulation pairs based on the 1-distance and filtered the pairs whose similarity was less than 0.6. In addition, a feed forward loop (FFL) is an important building block of regulatory mechanisms and is related to the development of tumors, in which one TF *M* regulates another TF *N*, and *M* and *N* jointly regulate their target gene *G*. Therefore, we identified FFLs in the regulatory network to construct the final specific regulatory network.

Ultimately, the specific regulatory network consisted of 121 TFs, 439 target genes and 2081 regulatory pairs. There were two categories of edges in the specific regulatory network: 394 TF-TF pairs and 1687 TF-target gene pairs. Each edge that corresponded to two nodes in the network had a tumor-specific regulation relationship, and the similarity of the edge represented the degree of regulation between the two nodes.

*ETS1* was the node with the highest degree in the specific regulatory network. *ETS1* is a protein-coding TF that can act as an activator or inhibitor of multiple genes in a variety of different cellular environments. Moreover, annotations of Gene Ontology (GO) related to *ETS1* indicate that the gene participates in various biological functions, such as cell senescence, apoptosis, and cell development, and plays an important role in the occurrence of diseases. *ETS1* upregulates the expression of the integrin α5 subunit and mediates intracellular signal transduction and invasion processes, leading to the occurrence of malignant glioma [50].

### Cell type identification

We identified 121 regulation meta modules (RMMs) in the specific regulatory network, and then the RMMs were considered as single-cell features to obtain the specific regulation expression matrix. Next, we used hybrid clustering to identify the cell types, and reproducibility-optimized test statistic (ROTS) method [44] was used to identify the gene biomarkers of different cell types. The process of cell type identification fully considered the differences among tumor samples from malignant cells and the effect of transcriptional regulatory mechanisms on gene expression profiles. The resulting 6 cell types identified are shown in Table 2 and were named cell types A to F.

**Table 2:** Results of cell types identification of glioma

Cell type	A	B	C	D	E	F
Num. of cells	136	983	186	214	238	2287
Num. of gene biomarkers in cell type*	229	29	11	388	4	49

*ROTS is used in obtaining the gene biomarkers

To further analyze the functional and biological significance of different cell types, we performed enrichment analysis for gene biomarkers in each cell type. Enrichment analysis was used as the priori knowledge such as gene annotation to classify a group of genes, and the classification results could help explore whether these genes had certain functions in common and understand the role of genes in life activities. In this study, the Metascape tool [45] was used for analysis.

**Fig. 4:**
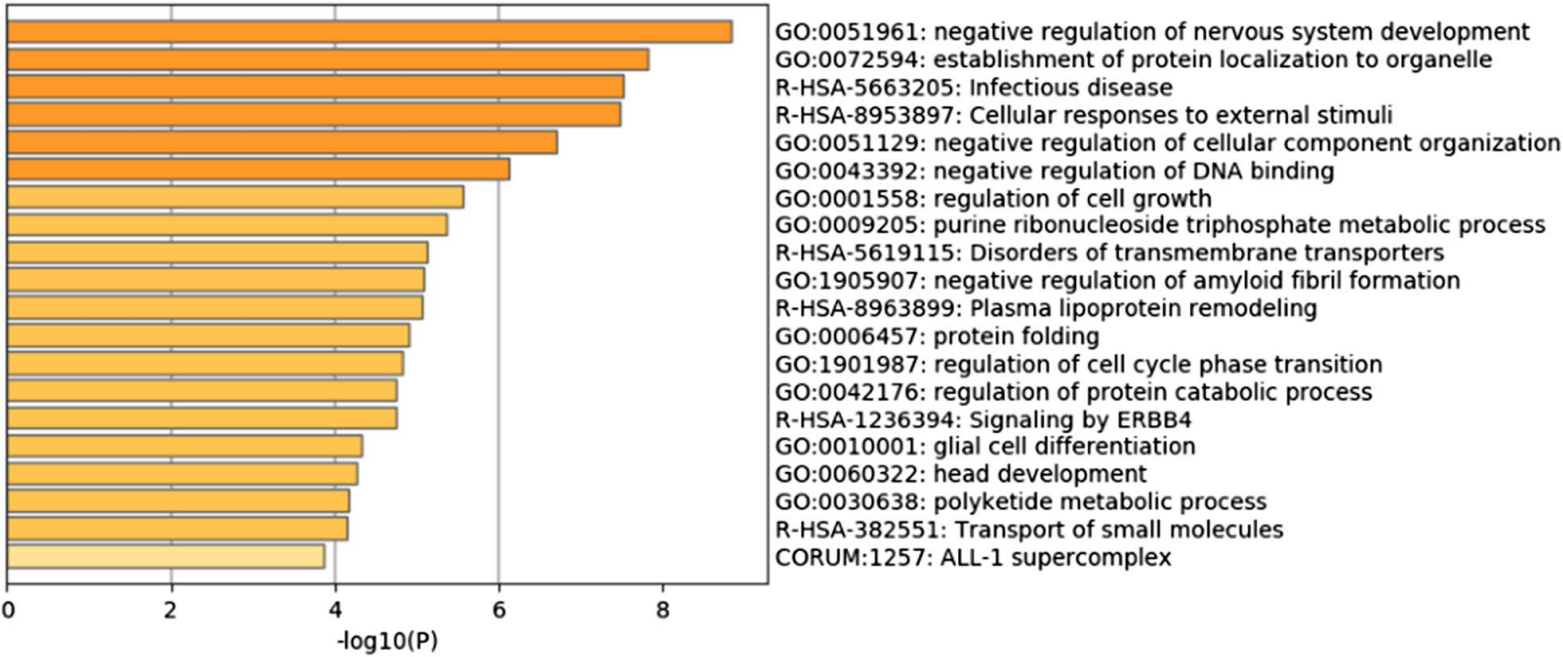
Enrichment analysis results of Metascape of cell type A. The genes were mainly enriched in biological functions such as glial cell differentiation, the ERBB4 signaling pathway, and nervous system development

**Fig. 5:**
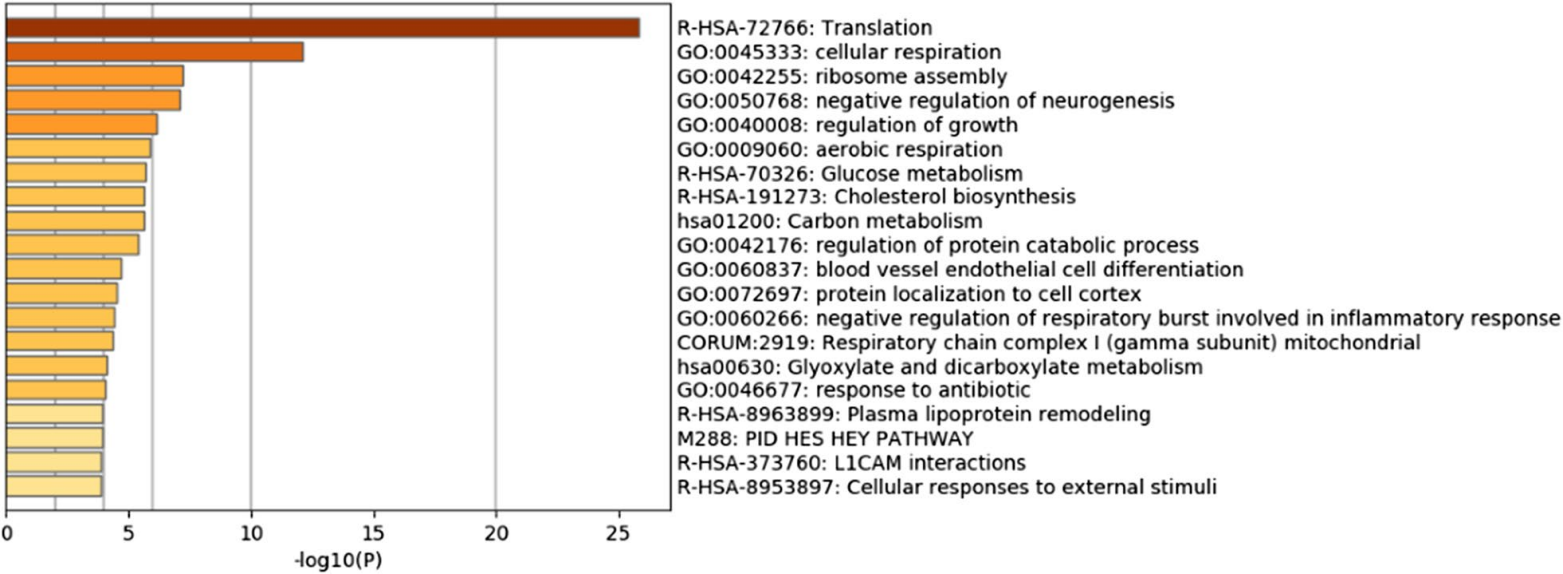
Enrichment analysis results of Metascape of cell type D. The genes were mainly enriched in a number of biological functions related to cellular respiration, including aerobic respiration and the negative regulation of respiration involving inflammation

Figures 4 and 5 show the enrichment analysis results for cell types A and D, respectively. The more depth the color of the bar is, the greater the enrichment of the gene. For cell type A, the genes were mainly enriched in biological functions such as glial cell differentiation, the ERBB4 signaling pathway, and nervous system development. Among them, ERBB4 belongs to the ERBB receptor family and plays an important role in the development of the nervous system, and the ERBB growth factor receptor is considered to be a key signaling pathway for many human tumors, including glioma [51]. For cell type D, the genes were mainly enriched in a number of biological functions related to cellular respiration, including aerobic respiration and the negative regulation of respiration involving inflammation. Hypoxia could lead to increased aggressiveness of tumors, and tumor growth, metastasis and resistance to drug treatment greatly improved in the hypoxic microenvironment. There was also some evidence that the hypoxic response plays a key role in the behavior of glioma cells, which is very important for personalized treatment of patients with glioma [52].

The four most enriched entries of cell types B, C and F are shown in Table 3. Table 3 shows that cell type B was mainly involved in a variety of cellular metabolic activities; cell type C was mainly related to apoptosis, inhibition of cell growth and other biological functions; and cell type F was associated with biological functions related to multiple ribosomal proteins. Cell metabolism, apoptosis, and disturbance of ribosomal proteins could cause many complex diseases, including cancer. In addition, since only 4 gene biomarkers were found in this cell type, there were no related enrichment items. Howerer, two of these genes are known cancer-related genes, indicating that cell type E may also be related to the development of glioma.

**Table 3:** Enrichment analysis results of gene sets in cell type B, C, and F

Cell type	Enriched item	Function*	log10(p)^#^
Cell type B	R-HSA-71291	Metabolism of amino acids and derivatives	− 5.3
Cell type B	R-HSA-69206	G1/S transition	− 3.3
Cell type B	GO:0010565	Regulation of cellular ketone metabolic process	− 2.9
Cell type B	GO:0032787	Monocarboxylic acid metabolic process	− 2.1
Cell type C	GO:0072331	Signal transduction by p53 class mediator	− 3.7
Cell type C	GO:0097193	Intrinsic apoptotic signaling pathway	− 3.6
Cell type C	GO:0071363	Cellular response to growth factor stimulus	− 2.5
Cell type C	GO:0080135	Regulation of cellular response to stress	− 2.4
Cell type F	R-HSA-72689	Formation of a pool of free 40S subunits	− 35.0
Cell type F	R-HSA-72695	Formation of the ternary complex, and subsequently, the 43S complex	− 15.1
Cell type F	CORUM:5380	TRBP containing complex (DICER, RPL7A, EIF6, MOV10 and subunits of the 60S ribosomal particle)	− 8.8
Cell type F	GO:0042255	Ribosome assembly	− 5.1

*Function description for each enriched item

^#^log *p* value for each enriched item

Metascape enrichment analysis indicated that each cell type had unique functionality, and the gene biomarkers may be closely related to the occurrence and treatment of glioma.

### Candidate tumor gene biomarker analysis of glioma

Assuming that KGGs are specifically expressed in glioma, we used the first principal component method to calculate the tumor feature vector (TEV) for all KGGs based on bulk RNA-seq gene expression data. TEV was a linear combination of all KGG expression vectors, which could represent the expression level of all KGGs. In addition, gene biomarkers of cell types reflected the biological function of glioma and were likely to be the causative molecules of glioma. Therefore, we took all of these genes as candidate genes and calculated the Pearson correlation coefficient (PCC) between the candidate gene and TEV. The greater the absolute PCC value is, the stronger the relationship between the candidate gene and glioma. The absolute PCC value was used as the correlation between the candidate gene and TEV. We analyzed the top 20 genes in detail and defined them as candidate tumor gene biomarkers of glioma (Table 4). Statistical results showed that the correlations between two candidate tumor gene biomarkers (*ATP6V0B* and *GUK1*) were extremely strong, with correlations greater than 0.8. The correlations of the remaining 18 candidate tumor gene biomarkers were strong (between 0.6 and 0.8).

**Table 4:** Candidate tumor gene biomarkers of glioma

Ranking	Candidate tumor gene biomarkers	Score*	PubMed ID
1	*ATP6V0B*	0.825678	–
2	*GUK1*	0.816174	11156382
3	*MRPL20*	0.774609	–
4	*RAB30*	0.718324	24080485
5	*NDUFS5*	0.712145	31747975
6	*TMEM160*	0.710739	–
7	*ZNF195*	0.70465	–
8	*DDRGK1*	0.69812	–
9	*ARF5*	0.691213	–
10	*MRPL41*	0.690842	28351326
11	*NDUFA1*	0.685764	29211022
12	*GOLIM4*	0.683062	–
13	*COX5B*	0.682042	29180880
14	*NDUFA13*	0.681426	31747975
15	*NDUFB8*	0.675849	29928884
16	*NCOA4*	0.673071	–
17	*ARL2*	0.659971	29843637
18	*SDHB*	0.645852	29890994
19	*ROGDI*	0.645402	–
20	*BMPR1A*	0.643674	26683138

*–* No supported publiccations were found in PubMed

*Score indicates that the Pearson correlation coefficient (PCC) between the candidate gene and tumor feature vector (TEV)

Additionally, 11 out of the 20 candidate tumor gene biomarkers were confirmed by relevant medical literature that they had a direct or indirect relationship with glioma (Table 4), which showed that the identified candidate tumor gene biomarkers were reliable to a certain extent. Some candidate genes are found to be associate with cancer in GeneCards, a database of human genes that provides genomic, proteomic, transcriptomic, genetic and functional information on all known and predicted human genes [53]. For example, the protein encoded by *GUK1* is thought to be a good target for cancer chemotherapy. *ZNF195* is located near the centromeric border of chromosome 11p15.5, next to an imprinted domain that is associated with maternal-specific loss of heterozygosity in Wilms' tumors. Chromosomal translocations between *NCOA4* and the ret tyrosine kinase gene are associated with papillary thyroid carcinoma. Sporadic and familial mutations in *SDHB* result in paragangliomas and pheochromocytoma, and support a link between mitochondrial dysfunction and tumorigenesis. In addition, we found that four (*NDUFS5*, *NDUFA1*, *NDUFA13*, and *NDUFB8*) of the candidate tumor gene biomarkers belong to the NADH ubiquinone oxidoreductase subunit gene family. This gene family plays a key role in the transfer of NADH to the respiratory chain. The protein encoded by these four genes is a subunit of the NADH:ubiquinone oxidoreductase (complex I), and is the first enzyme complex in the electron transport chain. The protein binds the signal transducers and activators of transcription 3 (STAT3) transcription factor, and can function as a tumor suppressor. NADH is the reduced state of nicotinamide adenine dinucleotide and is mainly involved in the metabolism of matter and energy in cells, which plays a key role in maintaining cell growth and differentiation. The studies of Yuan et al. [54] and Trinh et al. [55] showed that NADH is regarded as a new marker to classify glioma cancer cells. Therefore, we inferred that the NADH ubiquinone oxidoreductase subunit gene family may be closely related to glioma.

### Survival analysis of candidate tumor gene biomarkers of glioma

**Fig. 6:**
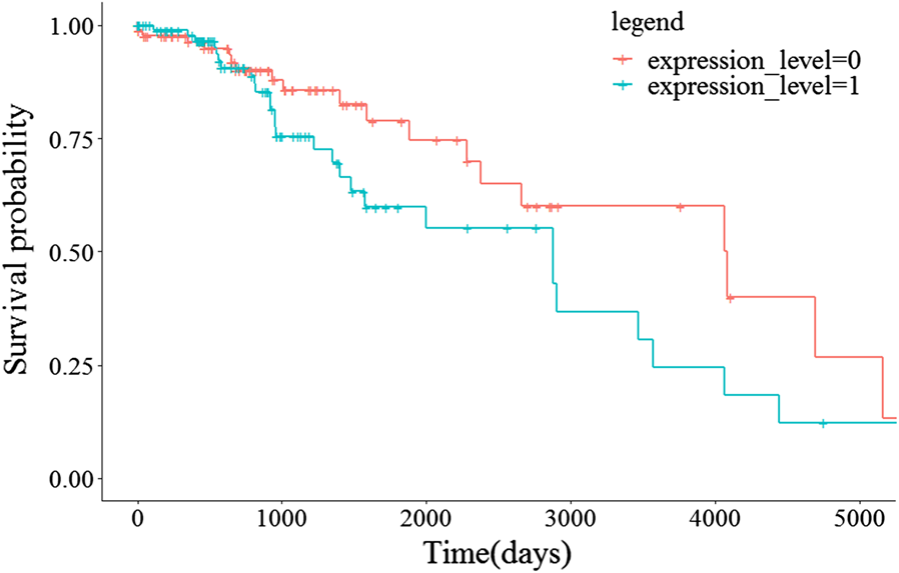
KM survival analysis results of ATP6V0B in OS survival data. Glioma samples were divided into a high expression group (expression_level = 1) and a low expression group (expression_level = 0), according to the median expression value of the candidate tumor gene biomarker in bulk RNA-seq profiles. The red curve and the blue curve represent the survival curves of the low and high expression sample groups, respectively

To further explore the effect of the expression level of candidate tumor gene biomarkers on the prognosis of gliomas, overall survival (OS) data were used for survival analysis. Specifically, we used specific survival time for the Kaplan–Meier (KM) survival curve analysis. Figure 6 shows the results of the KM survival analysis of the most highly correlated tumor gene biomarker (*ATP6V0B*). Glioma samples were divided into a high expression group (expression_level = 1) and a low expression group (expression_level = 0), according to the median expression value of the candidate tumor gene biomarker in bulk RNA-seq profiles. The red curve and the blue curve represent the survival curves of the low and high expression sample groups, respectively. Analysis of the downward trend of the KM curve in Fig. 6 revealed that the interval between the survival curves of the high and low expression samples of ATP6V0B was quite obvious, and the samples with high expression exhibited the worse prognosis.

For each candidate tumor gene biomarker, we divided samples into a high expression group and a low expression group and analyzed the downward trend of the KM survival analysis between the two curves. The interval between curves of the two groups clearly indicated that the gene expression level affected the survival of patients with glioma. The survival curve of the high expression sample group was below that of the low expression sample group, indicating that the patients with high gene expression had a worse prognosis, whereas the patients with low gene expression of the gene had a worse prognosis. Table 5 summarizes the KM survival analysis results of candidate tumor gene biomarkers. There were 5 genes (*ATP6V0B*, *MRPL20*, *NDUFS5*, *DDRGK1*, and *SDHB*) with high expression levels, and the prognosis of the patient was worse. In addition, there were 5 genes (*GUK1*, *ZNF195*, *MRPL41*, *NDUFA13*, and *BMPR1A*) in which the expression level was low, and the prognosis of the patient was worse.

**Table 5:** KM survival analysis results of candidate tumor gene biomarkers

Expression level	Candidate tumor gene biomarkers				
High expression	*ATP6V0B*	*MRPL20 *	*NDUFS5 *	*DDRGK1*	*SDHB*
Low expression	*GUK1*	*ZNF195*	*MRPL41*	*NDUFA13*	*BMPR1A*

The prognosis of the patient was worse when high expression genes are in high expression levels or low expression genes are in low expression levels

The experimental results of survival analysis showed that the identified candidate tumor gene biomarkers were reliable and had a strong correlation with glioma, and these genes could provide clues for the diagnosis and treatment of patients with gliomas and further help to understand the molecular mechanisms of glioma.

## Discussion

Due to the batch effect or other factors, the individual differences in malignant cells among tumor samples are strong. The identification of cell types can essentially be regarded as an unsupervised clustering process. If cluster analysis of malignant cells based on single-cell expression profiles is performed directly, malignant cells from the same individual will often cluster together, and the clustering results may not reflect the tumor cell types. However, there will be consistency among different samples of the tumor. To identify the genes that were coexpressed in the tumor and the genes that expressed heterogeneity in different tumor samples, we analyzed the cell-specific network from the single-sample level to identify tumor consensus genes and then combined the transcriptional regulatory pairs with multisample cell expression data to identify glioma cell types. Since the gene biomarkers of the cell types have a strong correlation with glioma, we used the correlation assessment method to predict the candidate tumor gene biomarkers that are highly related to glioma. From the analysis results, we concluded that the identified candidate tumor gene biomarkers had a strong correlation with glioma. The research results may be helpful for the diagnosis and treatment of patients with glioma, but these predicted candidate tumor gene biomarkers should be verified by further biological experiments. Of course, there are some problems in our study. For example, the results were heavily affected by the input gene expression data and noise in the data. In the future, we will integrate more omics data to perform further analyses, such as DNA methylation, noncoding RNA regulation, and protein interactions.

## Conclusion

In this study, we have proposed a new framework for identifying candidate tumor gene biomarkers based on single-cell gene expression profiles and transcriptional regulation pairs. The framework mainly contains four steps: preprocessing of single-cell gene expression data, identification of tumor consensus genes, identification of tumor cell types, and identification of candidate tumor gene biomarkers. We have shown the framework’s performance by exploring the molecular mechanisms of glioma. For glioma, 6 cell types and 20 candidate tumor gene biomarkers were identified. The Metascape enrichment analysis showed that the cell types had significant functionality, and the analysis of candidate tumor gene biomarkers showed that it had a strong correlation with glioma. In addition, recent relevant studies have also shown that some candidate tumor gene biomarkers were recognized as targets of glioma, and 4 genes (*NDUFS5*, *NDUFA1*, *NDUFA13*, and *NDUFB8*) of the candidate tumor gene biomarkers belonged to the NADH ubiquinone oxidoreductase subunit gene family, indicating that this gene family may have a strong correlation with glioma. These findings contributed to the clinical diagnosis, therapeutic drug development, and pathological mechanisms of glioma.

## Data Availability

The datasets generated during and/or analysed during the current study are available as follow: Single-cell gene expression data of glioma is available in CancerSEA with dataset ID EXP0062 and downloaded from GEO, (https://www.ncbi.nlm.nih.gov/geo/query/acc.cgi?acc=GSE70630) [30]. Transcriptional regulation pairs are available in HTRIdb, (http://www.lbbc.ibb.unesp.br/htri/pagdown.jsp) [32], and TRRUST, (https://www.grnpedia.org/trrust/data/trrust_rawdata.human.tsv) [33]. Known cancer-related genes are available in OMIM, (https://www.omim.org/search?index=geneMap&start=1&sort=chromosome_number+asc%2C+chromosome_sort+asc&search=glioma&limit=10) [34], COSMIC, (https://cancer.sanger.ac.uk/cosmic/download) [35], and Bailey’s research, (https://doi.org/10.1016/j.cell.2018.02.060) [36]. Bulk RNA-seq and clinical data from glioma is available in TCGA, (https://portal.gdc.cancer.gov/projects/TCGA-GBM) [37].

## Abbreviations

KGG: Known glioma-related gene
TF: Transcription factor
FFL: Feed forward loop
RMM: Regulation meta module
PCC: Pearson correlation coefficient
OS: Overall survival
